# Do thyroid peroxidase antibodies influence risk of cardiovascular diseases before and after treatment of hyperthyroidism?

**DOI:** 10.1186/1756-6614-6-S2-A10

**Published:** 2013-04-05

**Authors:** Anna Brona, Anna Bohdanowicz-Pawlak, J Jakubowska, Diana Jędrzejuk, Andrzej Milewicz

**Affiliations:** 1 Department of Endocrinology, Diabetology and Isotope Therapy, Medical University of Wroclaw, Wroclaw, Poland

## 

We have evaluated how levels of predictors of the cardiovascular risk such as: glucose, insulin, total cholesterol, high-density cholesterol, low-density cholesterol and triglycerides as well as adiponectin, fibrinogen, D-Dimers and CRP changed 24-28 weeks after treatment of hyperthyroidism in women with low and high titer of antibodies, and if their levels were different in these groups before and after treatment of hyperthyroidism. We compared also fT4 level before and after treatment in study groups.

We investigated 35 postmenopausal women (non-smoking, aged 51-69 years) with subclinical and overt hyperthyroidism. We divided them into two groups according to titer of thyroid peroxidase antibodies: with low titer of antibodies (27 women) and high titer of antibodies (8 women).

Statistical analysis revealed no difference in lipids profile, coagulation-fibrinolytic system, glucose, insulin, CRP and adiponectin level (before and after treatment) between groups with low and high titer of antibodies. No difference in fT4 level before and after treatment was found. In women with low titer of antibodies significant decrease was observed only in fT4 level (20.38 vs. 13.23 ng/ml). Levels of other factors did not differ before and after treatment. Triglycerides level decreased (133.00 vs. 89.13 mg/dl) and CRP level increased (2.95 vs. 4.48 mg/l) significantly after treatment in women with high titer of antibodies.

Data suggest that it is more difficult to become euthyroid for women with hyperthyroidism and high titer of antibodies (there is no significant difference between fT4 level before and after treatment). Changes in metabolic profile and increase in inflammatory process are observed in women with hyperthyroidism and high titer of antibodies.

